# Surgical Treatment for a Giant Solitary Plasmacytoma with Skull Erosion

**DOI:** 10.7759/cureus.3535

**Published:** 2018-11-01

**Authors:** Yi-Hsuan Kuo, Wen-Cheng Huang, Jau-Ching Wu

**Affiliations:** 1 Neurosurgery, Taipei Veterans General Hospital, Taipei, TWN

**Keywords:** solitary plasmacytoma, solitary plasmacytoma of skull

## Abstract

Solitary plasmacytoma of the skull, a single malignant monoclonal plasma cell proliferation without systemic involvement, is rare and often misdiagnosed by radiological examinations only. In this article, the authors presented a 40-year-old man who had a painless protruding mass over the midline of the posterior head region. A brain magnetic resonance imaging (MRI) revealed an enhanced mass lesion over the midline of the parieto-occipital region with skull erosion. Under the tentative diagnosis of meningioma, craniectomy was performed with en bloc tumor resection, and the skull defect was replaced by cranioplasty with bone cement. The final histopathological report revealed plasmacytoma without evidence of multiple myeloma. No further adjuvant radiotherapy was arranged for the patient. The postoperative course was uneventful within a one-year follow-up period. For the skull solitary plasmacytoma, there was no strong evidence that adjuvant radiotherapy was necessary after the primary surgery. Surgical intervention with total tumor resection is an effective option for the patient with solitary plasmacytoma of the skull.

## Introduction

Solitary bone plasmacytoma, a single lesion with malignant proliferation of monoclonal plasma cells in the bone, is rare and affects less than 5% of patients with plasma cell myeloma [1-3]. The prevalence of plasmacytoma is dominant in males, and the average age at presentation is approximately 55 years. The most common location is the axial skeleton, particularly in vertebrae [1, 3]. Solitary plasmacytoma of the skull is rare. The preoperative diagnosis is easily misleading if only based on the information of brain magnetic resonance imaging (MRI) [2, 4-6]. Because of its rarity, there weren’t many evidences about the effectiveness of various treatment options. In this study, we presented a rare case of solitary plasmacytoma of the skull that was diagnosed as meningioma preoperatively. The patients had an uneventful one-year follow-up after surgical intervention with total tumor removal. The related literature was reviewed and is discussed in this article for treatment of the solitary plasmacytoma of the skull.

## Case presentation

**Figure 1:**
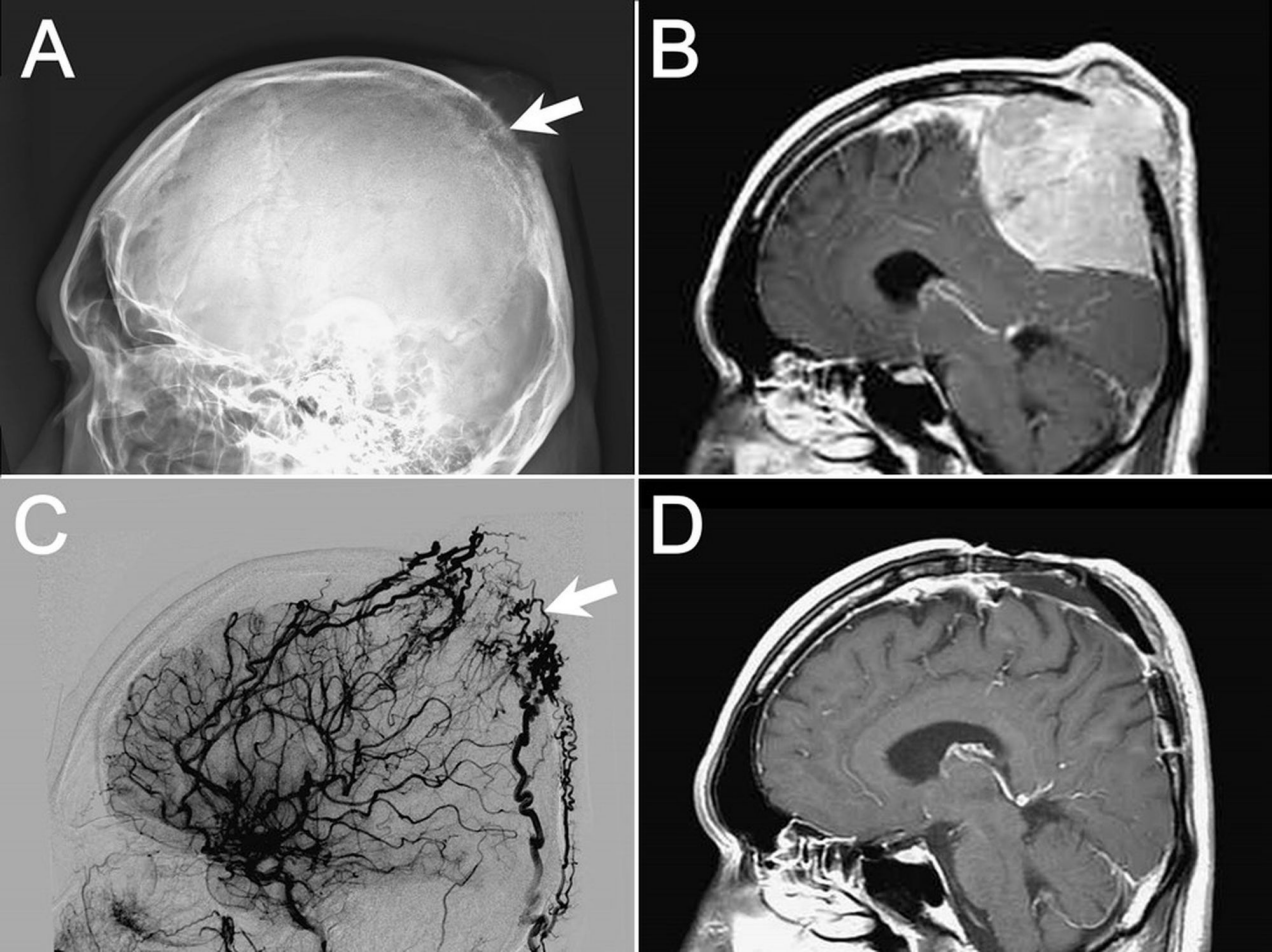
Preoperative and postoperative images. Figure 1A: Skull erosion (arrow) was found in the plain film. Figure 1B: In preoperative magnetic resonance imaging (MRI) (gadolinium-enhanced T1-weighted sagittal view), a large parasagittal tumor was seen with homogenous contrast enhancement and bone erosion, causing both intracranial and extracranial mass effect. Figure 1C: The tumor was highly vascularized (arrow) as demonstrated by angiography. Figure 1D: Postoperative MRI at one year showed no evidence of residual or recurrent tumor.

A 40-year-old man had noticed a painless protruding mass under the midline parietal-occipital scalp for approximately one year. On plain radiograph (Figure 1A), there was erosion of the skull bone underneath. The gigantic mass was better depicted by a magnetic resonance imaging (MRI), which demonstrated a large parasagittal tumor with homogenous contrast enhancement (Figure 1B, Gadolinium-enhanced T1-weighted MRI) that was considered meningioma.

Hypervascularity of the lesion was suspected based on the MRI with strong enhancement. Angiography revealed dense stains with distorted vessels over the same location (Figure 1C). Embolization prior to craniotomy was performed. During surgery, the tumor was noted to be dark-reddish in color and rubbery in texture; it had invaded through the skull but could be easily separated from the dura. The tumor was completely removed along with the invaded skull and bone cement was used for cranioplasty.

**Figure 2:**
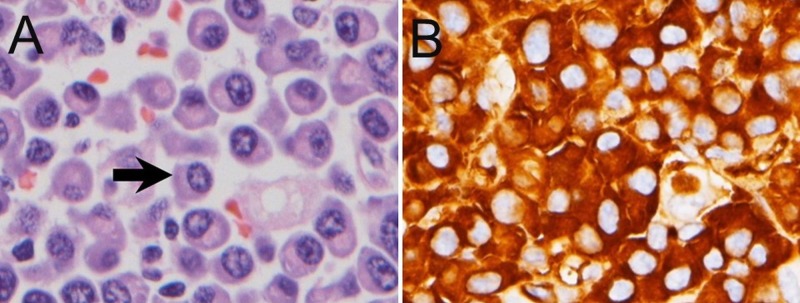
Histopathology of the tumor. Figure 2A: Hematoxylin and eosin stain, atypical plasma cells with eccentric nuclei and perinuclear halo were found (400X, arrow). Figure 2B: Positive Lambda immunostain, 400X.

Histopathology demonstrated plasmacytic type plasmacytoma with positive lambda stain (Figures 2A, 2B). Systemic oncological evaluations detected no evidence of residual tumor or other skeletal involvement, no tumor cells by bone marrow biopsy, no anemia, and no hypercalcaemia or renal impairment due to plasma cell dyscrasia. Radiotherapy was not performed because of total tumor resection. The postsurgical period was smooth and there was no laboratory or radiologic evidence of recurrence or systemic progression after the patient was regularly followed for one year (Figure 1D).

## Discussion

In this study, we presented a 40-year-old man with a solitary plasmacytoma at the midline parietal-occipital region of the skull. The patient was treated by en bloc tumor resection and cranioplasty by bone cement. Radiotherapy was not performed because there was no residual tumor, and chemotherapy was also not arranged since there was no systemic involvement.

Solitary plasmacytoma of the skull is rare. The image features of solitary plasmacytoma of the skull included osteolytic lesion with well-defined margin, non-sclerotic borders in a computed tomography (CT) scan, and typically T1 hypointense and T2 hyperintense in MRI with enhancement after contrast injection [7]. Because of its rarity, it is easily misdiagnosed as meningioma with skull invasion preoperatively, if it extended intracranially [2, 4-6].

Unlike multiple myeloma, there is no systemic involvement in patients with solitary bone plasmacytoma. There were several recommended diagnostic criteria for solitary bone plasmacytoma, including single area of bone destruction due to clonal plasma cells; normal marrow without clonal disease; normal results on a skeletal survey and magnetic resonance imaging of the spine, pelvis, proximal femora, and humeri; no anemia, hypercalcemia, or renal impairment attributable to myeloma; and absent or low serum or urinary level of monoclonal protein and preserved levels of uninvolved immunoglobulins [1].

The related literature reviewed for the patients who had solitary plasmacytoma and received gross total resection including craniectomy and cranioplasty are listed in Table 1 [4, 8-13]. They all received gross total resection for the solitary skull lesion, which was proven as plasmacytoma histopathologically. Three of them received surgery alone and the remaining four had additional radiotherapy to the surgical field. There was no recurrence in either groups during follow-up except for one patient receiving radiotherapy whose postoperative condition was not available in the article. Considering the possible side effects such as secondary malignancy, radiation necrosis or radiation-induced vasculopathy, the necessity for radiotherapy after gross total resection of solitary plasmacytoma of the skull still needs more discussion.

**Table 1:** Patients with solitary plasmacytoma who received gross total resection and cranioplasty published in the English literature. GTR: gross total resection.

Study	Age	Gender	Location	Surgery	Cranioplasty (Material)	Radiation (Dose)	Follow-up	Recurrence
Arienta et al., 1987 [8]	64	F	Parietal	GTR	Yes (Tantalum wire mesh)	No	3 years	No
Du Preez et al., 1991 [4]	30	F	Frontotemporal	GTR	Yes	No	1.5 years	No
Barone et al., 1992 [9]	52	F	Frontal	GTR	Yes (autograft)	No	9 months	No
Matsuda et al., 1996 [12]	55	F	Temporal	GTR	Yes (autograft)	Yes (50 Gy)	2 years	No
Tanaka et al, 1998 [13]	55	M	Frontal	GTR	Yes	Yes (50 Gy)	7 months	No
Gürbüz et.ai., 2013 [10]	63	M	Parietooccipital	GTR	Yes (autograft)	Yes	-	-
Mankotia et al., 2017 [11]	36	M	Frontal	GTR	Yes (cement)	Yes	3 months	No

Definitive local radiotherapy is the treatment of choice for solitary bone plasmacytoma [2]. Treatment fields should be designed to encompass all disease shown by MRI or CT scanning and should include a margin of normal tissue [1]. Combining radiotherapy with surgery is increasingly considered as a treatment choice according to the tumor location [2]. As skull lesions usually cause intracranial mass effect and need surgical resection, craniectomy is a reasonable choice for solitary plasmacytoma of the skull. Subtotal resection should be followed by radiotherapy, and there is no role for chemotherapy if the patient has no evidence of multiple myeloma; however, there is paucity of discussion in the necessity of radiotherapy after gross total resection.

However, solitary plasmacytoma may present as an early manifestation of multiple myeloma in some cases. Therefore, frequent measurements of myeloma protein for at least six months after treatment are required to confirm disease radiosensitivity [1, 2].

## Conclusions

Solitary plasmacytoma is a rare solitary skull lesion with malignant monoclonal plasma cell proliferation. Gross total resection alone may be an effective treatment option, but solitary plasmacytoma of the skull requires continuous follow-up.
